# Molecular characterization of myelin basic protein a (*mbpa*) gene from red-bellied pacu (*Piaractus brachypomus*)

**DOI:** 10.1186/s43141-022-00296-6

**Published:** 2022-01-13

**Authors:** Juan Sebastian Cruz-Méndez, María Paula Herrera-Sánchez, Ángel Enrique Céspedes-Rubio, Iang Schroniltgen Rondón-Barragán

**Affiliations:** 1 Research Group in Immunobiology and Pathogenesis, Laboratory of Immunology and Molecular Biology, Faculty of Veterinary Medicine and Zootechnics, Universidad del Tolima, Santa Helena Highs, 730006299 Ibague, Tolima Colombia; 2 Research Group in Neurodegenerative Diseases, Faculty of Veterinary Medicine and Zootechnics, Universidad del Tolima, Santa Helena Highs, 730006299 Ibague, Tolima Colombia; 3 Laboratory of Immunology and Molecular Biology, Department of Animal Health, Faculty of Veterinary Medicine and Zootechnics, University of Tolima, Ibague, 730006299 Colombia

**Keywords:** Bioinformatics, Fish biomodel, Gene expression, Myelin basic protein, Phylogeny, Protein isoform

## Abstract

**Background:**

Myelin basic protein (MBP) is one of the most important structural components of the myelin sheaths in both central and peripheral nervous systems. MBP has several functions including organization of the myelin membranes, reorganization of the cytoskeleton during the myelination process, and interaction with the SH3 domain in signaling pathways. Likewise, MBP has been proposed as a marker of demyelination in traumatic brain injury and chemical exposure.

**Methods:**

The aim of this study was to molecularly characterize the myelin basic protein a (*mbpa*) gene from the Colombian native fish, red-bellied pacu, *Piaractus brachypomus*. Bioinformatic tools were used to identify the phylogenetic relationships, physicochemical characteristics, exons, intrinsically disordered regions, and conserved domains of the protein. Gene expression was assessed by qPCR in three models corresponding to sublethal chlorpyrifos exposure, acute brain injury, and anesthesia experiments.

**Results:**

*mbpa* complete open reading frame was identified with 414 nucleotides distributed in 7 exons that encode 137 amino acids. MBPa was recognized as belonging to the myelin basic protein family, closely related with orthologous proteins, and two intrinsically disordered regions were established within the sequence. Gene expression of *mbpa* was upregulated in the optic chiasm of the chlorpyrifos exposed fish in contrast to the control group.

**Conclusions:**

The physicochemical computed features agree with the biological functions of MBP, and basal gene expression was according to the anatomical distribution in the tissues analyzed. This study is the first molecular characterization of *mbpa* from the native species *Piaractus brachypomus*.

## Background

Oligodendrocytes of the central nervous system (CNS) and Schwann cells in the peripheral nervous system (PNS) generate the myelin sheath, covering axons to a proper propagation of neural signal at a high speed with a diminution of ionic leakiness, as well as preserving structures from degeneration [1–3]. In fish, myelination is conserved across species at a molecular and cellular level and also showed high conserved mechanisms with mammals [4]. Due to the rapid nerve conduction in myelinated neurons, myelin in different vertebrates have been related with to a rapid escape from predators [5]. Myelin membrane is a modified plasma membrane wrapped around nerve axon composed of lipids and proteins, from which myelin basic proteins (MBPs) are one of the most abundant [6]. MBPs are encoded by a large gene complex called genes of oligodendrocyte lineage (Golli), including 7 exons of the classic MBP [7]. Isoforms of classic MPB differ in size and are formed due to alternative splicing of a single mRNA transcript, presenting several different functions among them [7]. In this way, in fish, several MBP cDNAs have been reported [8, 9]. MBP is considered a key protein that forms a selective barrier that prevents the diffusion of cytosolic and membrane proteins to the myelin sheath region based on their size [10]. In addition, MBPs are necessary for the organization of the myelin membrane, reorganization of the cytoskeleton during the myelination process, and interaction with the SH3 domain in signaling pathways [10–12]. On the other hand, MBP has been proposed as a marker of active demyelination in traumatic brain injury (TBI), due to its response to structural damage, and the levels of its gene transcripts change after chlorpyrifos (CPF) exposure and anesthesia [13–15].

Furthermore, fish research has been widely used to improve our understanding of the complexity of the molecular and cellular mechanisms involved in human medical conditions, for new diagnostic and therapeutic tools, as well as to study the impact of environmental factors and pollution [16]. In the same way, teleost fish present advantages as models of brain regeneration, and currently, it has been demonstrated the importance of studying numerous species to comprehend different strategies that have evolved and could eventually be used to induce regeneration in non-regenerative species [17].

The red-bellied pacu, *Piaractus brachypomus*, is an endemic freshwater species used as a biomodel in pharmacological and immunotoxicology studies [18–21]. Thus, the aim of this study was to characterize the *mbpa* gene of the Colombian native fish red-bellied pacu (*Piaractus brachypomus*) through bioinformatic tools and in models of sublethal toxicity of chlorpyrifos, anesthesia with menthol and eugenol, and acute brain injury.

## Materials and methods

### Ethical approval

For the experimental procedures, the guidelines of the Local Bioethics Committee of the Research and Scientific Development Office of the University of Tolima were followed, based on Law 84/1989 and the Resolution 8430/1993, moreover complying with the established parameters for animal care and their use in research and teaching [22, 23].

### mbpa cDNA sequencing

Transcriptomic sequences of red-bellied pacu were obtained from brain cDNA sequencing carried out by the MinION sequencer method in our laboratory (Oxford Nanopore Technologies, UK). Reads were mapped with *Colossoma macropomum mbpa* gene (XM_036567606.1) as a reference using Geneious Prime software v2021.2.2 [24]. The resulting sequence was used to design specific primers for amplifying the complete open reading frame (ORF) of *mbpa* gene (Table 1). PCR was carried out in a ProFlex™ 3x32-Well PCR System thermal cycler (Thermo Fisher Scientific, USA) using GoTaq® Flexi DNA polymerase (Promega, Madison, USA). Amplification was performed in a reaction volume of 25 μL composed by 14,875 μL of distilled deionized water (ddH_2_O), 1 μL of cDNA template, 5 μL of Flexi Buffer 5x colorless GoTaq ® (Promega, USA), 1 μL of dNTPs (1.5 mM) (Invitrogen, USA), 1 μL of each primer (Table 1) at 10 pmol/μL (Macrogen, Korea), 1 μL of MgCl_2_ (25 mM) (Promega, USA), and 0.125 μL of GoTaq Flexi DNA polymerase (Promega, USA). The PCR amplification consisted of one initial denaturation cycle of 95°C for 3 min, followed by 35 cycles at 95°C for 30 s, 55°C for 30 s, and 72°C for 30 s, and a final extension of 72°C for 5 min. PCR products were confirmed by the agarose gel electrophoresis. The PCR product was sequenced by the Sanger method (Macrogen Inc., Korea). Reads were analyzed using BLAST (https://blast.ncbi.nlm.nih.gov) to identify the obtained sequences with the following parameters: database, non-redundant nucleotide sequences (nr); organism, all (by default); algorithm, BLASTn (nucleotide-nucleotide BLAST). Then, assembling was performed with Geneious Prime software v2021.2.2 [24].

**Table 1:** Sequences of primers for Sanger sequencing and qPCR

Gene	Sequence (5′-3′)		Amplicon size (pb)	Accession number
*mbpa complete ORF*	**F**	CCCAGGCTCAGAAGATCAGC	455	XM_036567606.1
	**R**	GGCTCTTTCCCGTCTCAGAAG		
*mbpa*	**F**	CTAGCACCTCAGGACAGAGC	188	MZ157122
	**R**	GTTCACATCTCCACGGCGTC		
ef1α*	**F**	ACTGAGGTCAAGTCTGTGGA	110	MK085759.1
	**R**	CCACGACGGATGTCTTTAA		

*Zapata et al. (2020)

### MBPa protein sequence analysis

From the nucleotide sequence, the amino acid composition of MBPa was predicted using Geneious Prime software v2021.2.2 [24], and both were reported to GenBank (Accession number MZ157122). Deduced protein was compared with reported sequences in the Protein database from NCBI through the BLASTp algorithm. The exons translated of MBPa from teleost fish were identified according to Nawaz et al. [1]. A search of conserved domains and protein motif was performed on InterproScan [25]. Intrinsically disordered regions (IDRs) were identified by MobyDB-lite [26], an integrated database on InterproScan. The primary structure was analyzed using the Expasy ProtParam tool, by computing the predicted molecular weight, theoretical isoelectric point (pI), the total number of both negatively as positively charged residues, instability index, and the grand average of hydropathicity (GRAVY) [27].

### MBPa multiple sequence alignment (MSA)

*mbpa* from *P*. *brachypomus* was used as a query for BLASTn search in order to find orthologous sequences from teleost fish. Twenty-three mRNA sequences were retrieved from the Gene NCBI database corresponding to *Thunnus maccoyii mbpa* (XM_042435722), *Coregonus clupeaformis mbpa* (XM_041902839), *Cheilinus undulatus mbpa* (XM_041809423), *Toxotes jaculatrix mbpa* (XM_041053828), *Notolabrus celidotus mbpa* (XM_034704637), *Perca fluviatilis mbpa* (XM_039821220), *Hippoglossus stenolepis mbpa* (XM_035182098), *Micropterus salmoides mbpa* (XM_038725849), *Sebastes umbrosus mbpa* (XM_037795862), *Pungitius pungitius mbpa* (XM_037474676), *Acanthopagrus latus mbpa* (XM_037092987), *Colossoma macropomum mbpa* (XM_036567606), *Etheostoma cragini mbpa* (XM_034892848), *Gymnodraco acuticeps mbpa* (XM_034235965), *Sander lucioperca mbpa* (XM_031295735), *Electrophorus electricus mbpa* (XM_027019538), *Anabas testudineus mbpa* (XM_026347403), *Amphiprion ocellaris mbp*a (XM_023271127), *Oncorhynchus mykiss mbpa* (XM_021571745), *Cyprinus carpio mbpa* (XM_019083323), *Pygocentrus nattereri mbpa* (XM_017700610), *Kryptolebias marmoratus mbpa* (XM_017440618), and *Fundulus heteroclitus mbpa* (XM_012851567). The complete ORF was translated, and then, MSA was performed with the 24 predicted amino acid sequences by ClustalW in Geneious Prime software v2021.2.2 [24].

### Phylogenetic analysis

Sequences of *mpba* (mentioned above) and *mbpb* mRNA from several teleost fish species were retrieved from the GenBank as follows: *Anabas testudineus mbpb* (XM_026370072), *Betta splendens mbpb* (XM_029128101), *Chelmon rostratus mbpb* (XM_041942705), *Coregonus clupeaformis mbpb* (XM_041871757), *Esox lucius mbpb* (XM_010885414), *Perca fluviatilis mbpb* (XM_039805415), *Periophthalmus magnuspinnatus mbpb* (XM_033981030), *Sander lucioperca mbpb* (XM_031278393), and *Simochromis diagramma mbpb* (XM_040013314). Additionally, sequences of *mbp* from cartilaginous fish taxon were chosen for the outgroup (*Heterodontus francisci* X17664, *Raja erinacea* REU44053, *Squalus acanthias* SAU44052). The longest available ORF was translated, and multiple sequence alignment was performed by using ClustalW in Geneious Prime software v2021.2.2 [24]. Then, the 36 amino acid sequences were used to represent evolutionary relationships of the fish MBP isoforms building a phylogenetic tree through the neighbor-joining method [28] with 10,000 replicates and the Jukes-Cantor genetic distance model [29] in Geneious Prime software v2021.2.2 [24].

### Sublethal exposure to chlorpyrifos for mbpa gene expression

Fingerlings of *P. brachypomus* with homogeneous weight (39 ± 2.4 g) were divided into two groups: fishes exposed to a concentration of 0.011 μg/L of chlorpyrifos [18] (*n* = 5), and a control group without CPF exposure (0 μg/L) (*n* = 5). Twenty-four hours before the experiment, fish feeding was suspended. Then, a semi-static tank system was used for the subsequent 3 days of the assay, with daily replacement of 50% of water and addition of CPF for maintaining the sublethal concentration. After the experimental period, fish were anesthetized using the hypothermic method [30] and sacrificed by cervical dislocation [31]. Brain tissues (olfactory bulb, optic chiasm, and telencephalon) were collected and kept in liquid nitrogen until subsequent use.

### Anesthesia assay for mbpa gene expression

Red bellied-pacu fingerlings (*n* = 10) with homogeneous weight (38.5 ± 3 g) were individually anesthetized by immersion in a glass tank with menthol (1R, 2S, 5R-2-isopropyl-5-methylcyclohexanol) (Farmacia Colony, Colombia) or eugenol (4-Allyl-2-methoxyphenol) (Proquident S.A., Colombia). Both anesthetic agents were dissolved by using absolute ethanol (Merck, Germany). The effective anesthesia concentration used was 50 mg/L for the menthol group (*n* = 5) and 40 mg/L for the eugenol group (*n* = 5) [32]. Fishes were euthanized by cervical dislocation when they reached general anesthesia state [31]. Then, samples of brain, gills, and liver tissues were collected and stored in liquid nitrogen until their use.

### Acute brain injury for mbpa gene expression

Experiments were performed following the brain injury procedures as described by Kishimoto et al. [33] and Schmidt et al. [34]. Six *Piaractus brachypomus* fingerlings with a body weight average of 38.5 ± 2.3 g were divided for the brain injury (*n* = 3) and a control group (*n* = 3). Before the acute cerebral injury, fishes were induced to stage III of anesthesia, described as total loss of swimming axis, by immersion in water at 2–4°C (hypothermic method) [30].

The injury was performed in the frontal region of the brain using a 000 gauge sterilized entomological needle, with a depth of 0.5 cm at a 45° angle. Then, fish were kept in a recovery tank (without anesthesia). After 2 h, the fishes were anesthetized as described previously and euthanized by cervical dislocation [31]. Individuals from control were under the same experimental conditions and procedures except for the brain puncture. Cerebral tissue was taken and kept in liquid nitrogen until their use.

### cDNA synthesis and quantitative real-time PCR (qPCR) assays

For all experimental procedures, tissues collected were homogenized completely by using of a F6/10 handheld homogenizer (Jingxin, China). RNA extraction was performed with TRizol reagent (Invitrogen, USA), the quality was measured by NanoDrop ™ One (Microvolume UV-Vis Spectrophotometer, Thermo Fisher Scientific, USA), and cDNA was synthesized using the High Capacity cDNA Reverse Transcription Kit (Thermo Fisher Scientific, USA). Expression of *mbpa* was carried out with gene-specific primers designed with Geneious Prime software v2021.2.2 [24] based on the reported sequence on the GenBank by our laboratory (Table 1). Basal tissue expression was assessed by qPCR in the brain, blood, intestine, stomach, kidney, muscle, spleen, heart, and gills. In addition, *mbpa* mRNA levels were assessed in two brain regions, being optic chiasm and olfactory bulb of red-bellied pacu. In the case of the treatments, the expression levels of *mbpa* gene were assessed in gills, liver, and brain tissue for anesthesia treatment and in brain tissue for sublethal chlorpyrifos exposure and acute brain injury. qPCR was carried out in a QuantStudio™ 3 Real-Time PCR System (Applied Biosystems, USA) using a total reaction volume of 20 μL as follows: 7 μL of ddH_2_O, 0.5 μL of each primer (Table 1) at 10 pmol/μL (Macrogen, Korea), 2 μL of tissue cDNA as template, and 10 μL of Luna® Universal qPCR Master Mix (New England Biolabs, USA). qPCR was set in Fast ramp mode, as recommended by the manufacturer, and PCR products were validated by melt-curve analysis. Relative gene expression was calculated using the 2^-ΔΔCt^ method [35] and elongation factor 1-α (*ef1α*) was used as a reference gene. Data were expressed as fold change.

### Statistical analysis

Data were analyzed by descriptive statistics and Shapiro-Wilk normality test. Basal relative gene expression of the tissues was performed using the Kruskal-Wallis test and Dunn’s test as a post hoc analysis. Additionally, basal expression of brain regions was assessed using a *t*-test. Relative gene expression for CPF exposure was evaluated by the Mann-Whitney test, while for the brain injury experiment, a *t*-test was performed; in the case of anesthesia experiment, gene expression was evaluated by the Mann-Whitney and *t*-test. Data were expressed as mean ± SEM. All statistical analyses were done with GraphPad Prism v 8.0 (La Jolla, USA), and differences were considered statistically significant when the *p*-value < 0.05.

## Results

### MBPa protein analysis and MSA

**Fig. 1:**
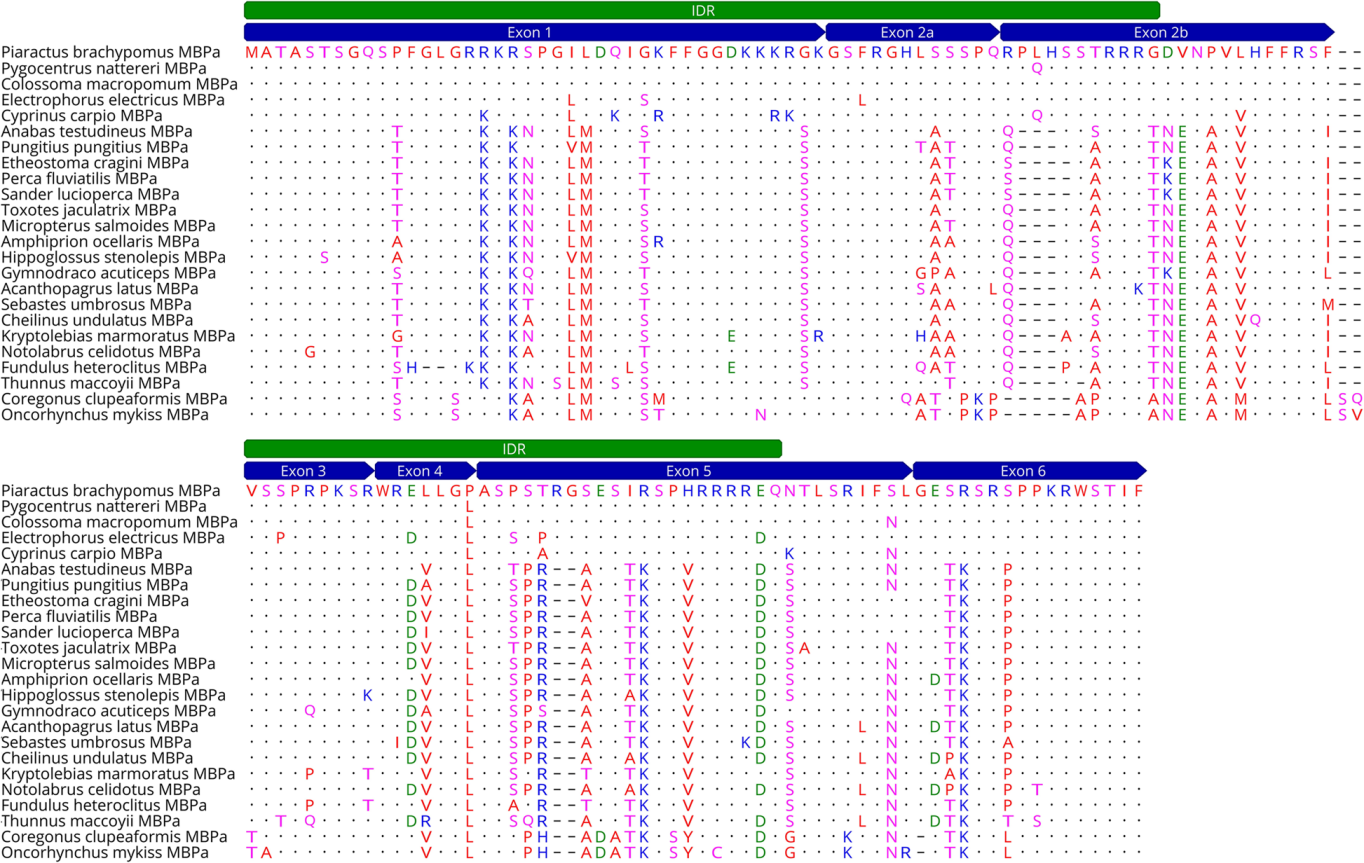
Amino acid sequence alignment of MBPa protein from 24 fish species. Residues are colored according to physicochemical properties as follows: blue (basic: R, K, and H), red (hydrophobic: A, V, F, P, M, I, L, W, G, and Y), green (acidic: D and E), and pink (polar: S, T, N, Q, and C). Dots represent the conservancy among sequences. IDRs (green annotations); seven exons of MBPa encoded (blue annotations)

The complete ORF of *mbpa* gene from *P*. *brachypomus* was detected by Sanger sequencing, with a total length of 414 bp that encodes a protein of 137 amino acids. Blastn search detected nucleotide identities with teleost fish *mbpa* ranging from 74.43 to 98.31%. Similarly, blastp from an amino acid sequence found between 64.67 and 98.54% identity with myelin basic proteins of several fish species. Seven exons were identified for the teleost MBPa protein, and InterproScan search detected a large region from the residues 1 to 132, corresponding to the Myelin basic protein family (PF01669) (Fig. 1). Additionally, amino acid regions from 1 to 63 and 78 to 114 were computed as IDRs by MobiDB-lite (Fig. 1). ProtParam tool calculated a molecular weight of 15457.53 Da (15.5 kDa) and theoretical pI of 12.28. Computed negatively (Asp+Glu) and positively (Arg+Lys) charged residues were 7 and 31, respectively. The instability index yielded a value of 115.27 and the GRAVY index was −1.036. MSA showed an identity among MBPa from *P. brachypomus* and 23 orthologous MBPa proteins from 61.15 to 98.54%. Although there was high conservation between the sequences, the highest identity was 98.54% with the species *Pygocentrus nattereri* and *Colossoma macropomum* (Fig. 1).

### Phylogenetic relationships

**Fig. 2:**
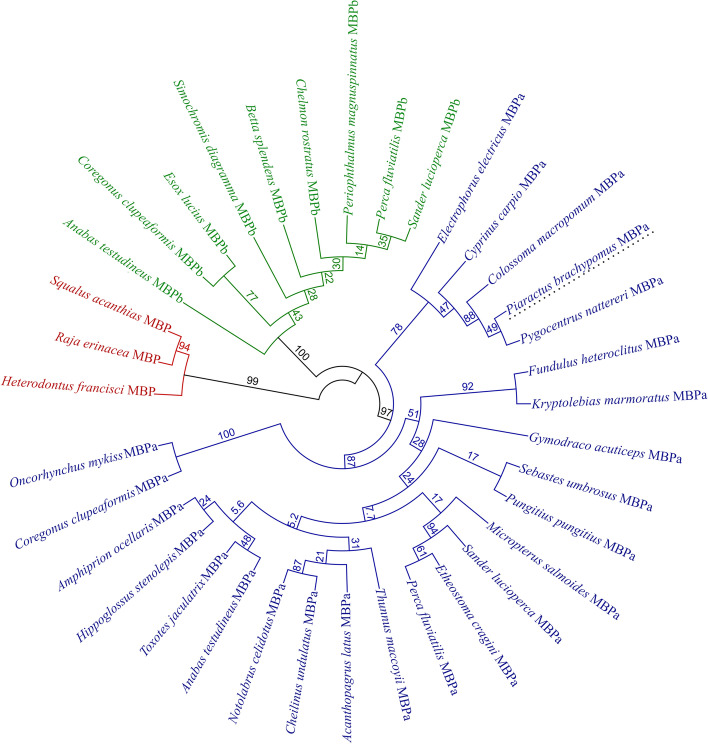
Phylogenetic tree of MBP from teleost and cartilaginous fish made by the neighbor-joining method. Red clade: MBP; green clade: MBPb; blue clade: MBPa. *Piaractus brachypomus* is noted in the dotted underline terminal taxon. Tree is composed of 36 amino acid sequences of MBP, MBPa, and MBPb translated from mRNA sequences retrieved from GenBank. Alignment was generated using ClustalW in Geneious Prime software 2021.2.2

The phylogenetic tree yielded three well-defined clades with a clear differentiation of MBP isoforms in fish (Fig. 2). Thus, the three sequences of cartilaginous fish belonging to Squaliformes, Rajiformes, and Heterodontiformes orders were grouped, out of the internal branches supported by 99% (red clade). Likewise, the nine MBPb sequences from teleost fishes were segregated in a cluster with 100% bootstrapping, conformed by the orders Perciformes, Salmoniformes, Esociformes, and Cichliformes (green clade). Similarly, the 24 MBPa proteins formed a group supported by 97%, including fish of the orders Gymnotiformes, Cypriniformes, Characiformes, Cyprinodontiformes, Perciformes, Scorpaeniformes, Gasterosteiformes, Pleuronectiformes, and Salmoniformes (blue clade). In the MBPa clade, *P*. *brachypomus* is closely related with other Characiformes fish in a group supported by 88%. Both MPBa and MPBb isoforms were recognized for *Anabas testudineus*, *Coregonus clupeaformis*, *Perca fluviatilis*, and *Sander lucioperca*.

### Basal gene expression of mbpa

**Fig. 3:**
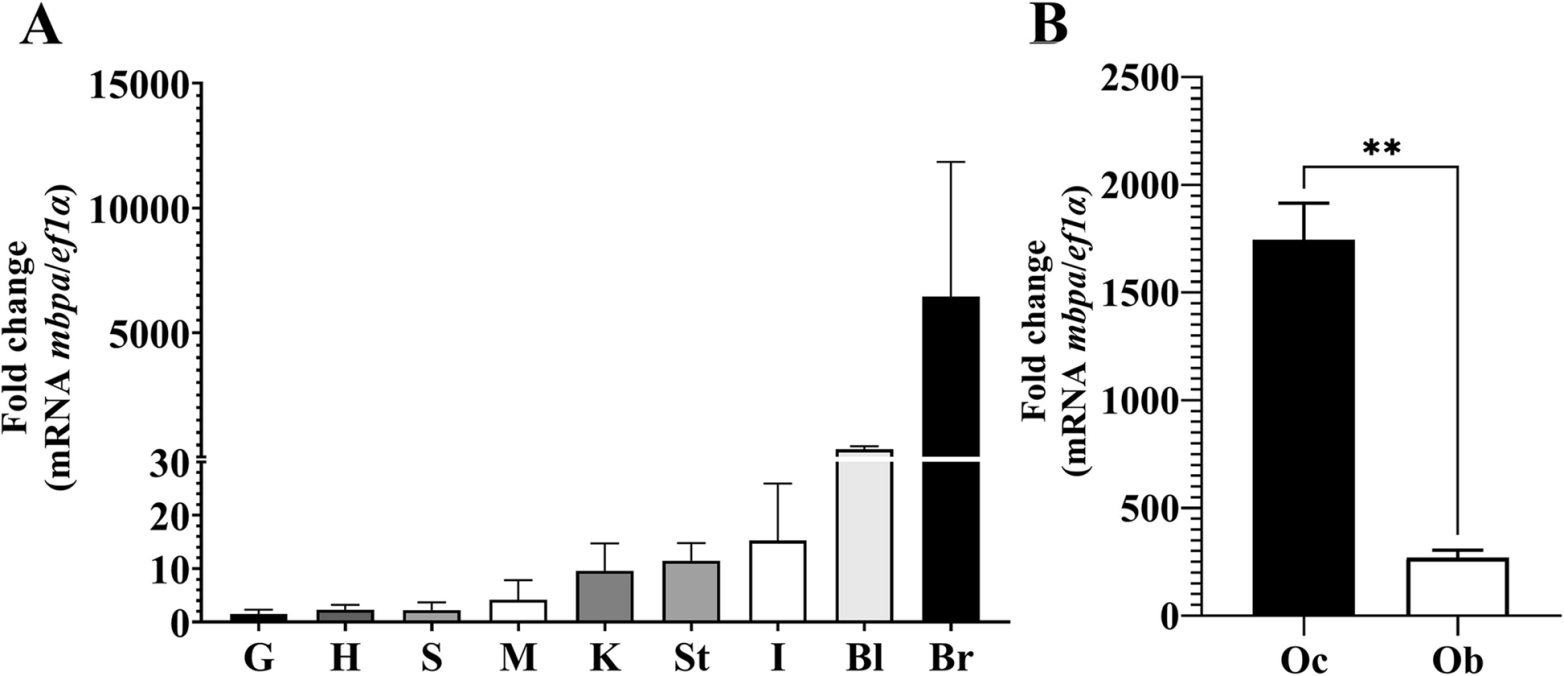
Relative gene expression of *mbpa* transcripts in *P*. *brachypomus* tissues. **A** Basal *mbpa* gene expression in several tissues. G gills, H heart, S spleen, M muscle, K kidney, St stomach, I intestine, Bl blood, Br brain. **B** Basal *mbpa* gene expression in two brain regions. Oc optic chiasm, Ob olfactory bulb. *ef1a* was used as a reference gene. **p < 0.01

*mbpa* transcripts were detected in all tissues and in the two brain regions from *P*. *brachypomus*. The highest tissue basal gene expression was found in the brain followed by the blood (Fig. 3A). A lower expression was detected in the intestine, stomach, kidney, muscle, spleen, heart, and gills (Fig. 3A). On the other hand, mRNA levels of *mbpa* were significantly higher in optic chiasm compared to the olfactory bulb (*p* < 0.0010) (Fig. 3B.).

### Gene expression of mbpa in three experimental models

**Fig. 4:**
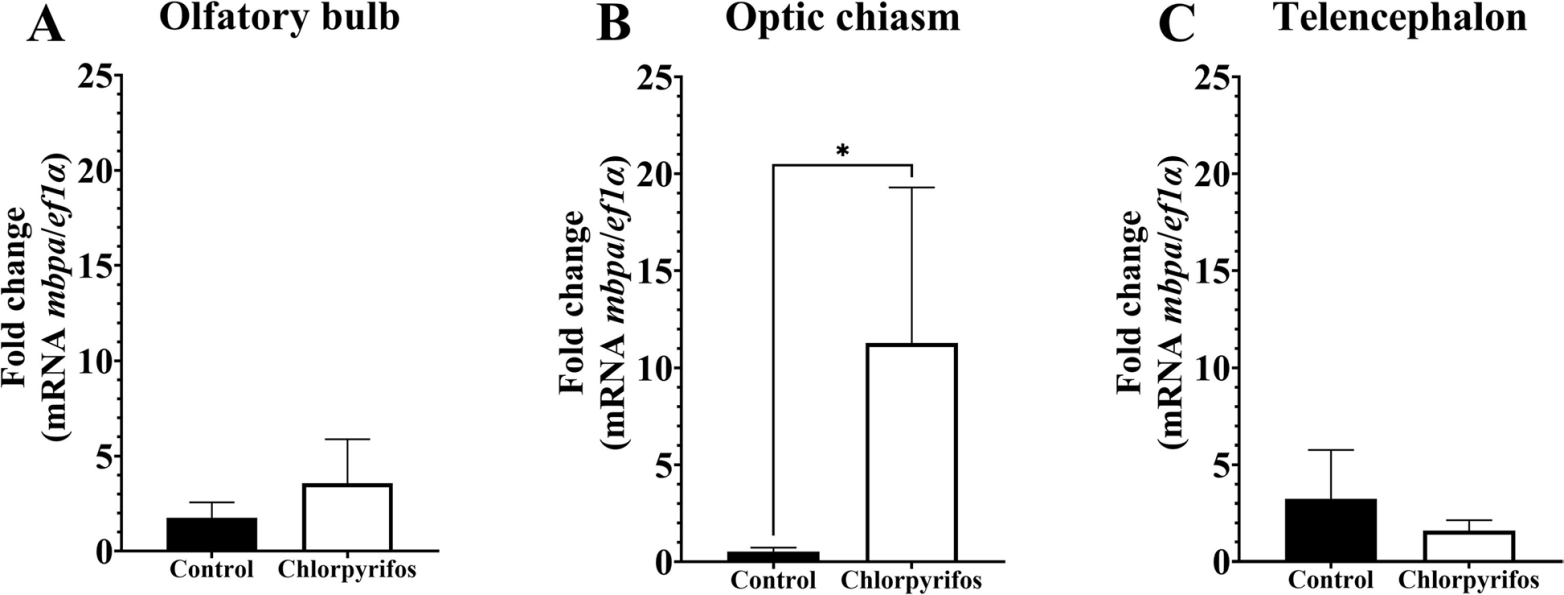
Relative gene expression of *mbpa* transcripts in *Piaractus brachypomus* after an exposition to Chlorpyrifos (0.011 μg/L). **A** *mbpa* levels in the olfactory bulb; **B** *mbpa* levels in optic chiasm; **C** *mbpa* levels in telencephalon. *ef1a* was used as a reference gene. *p < 0.05

**Fig. 5:**
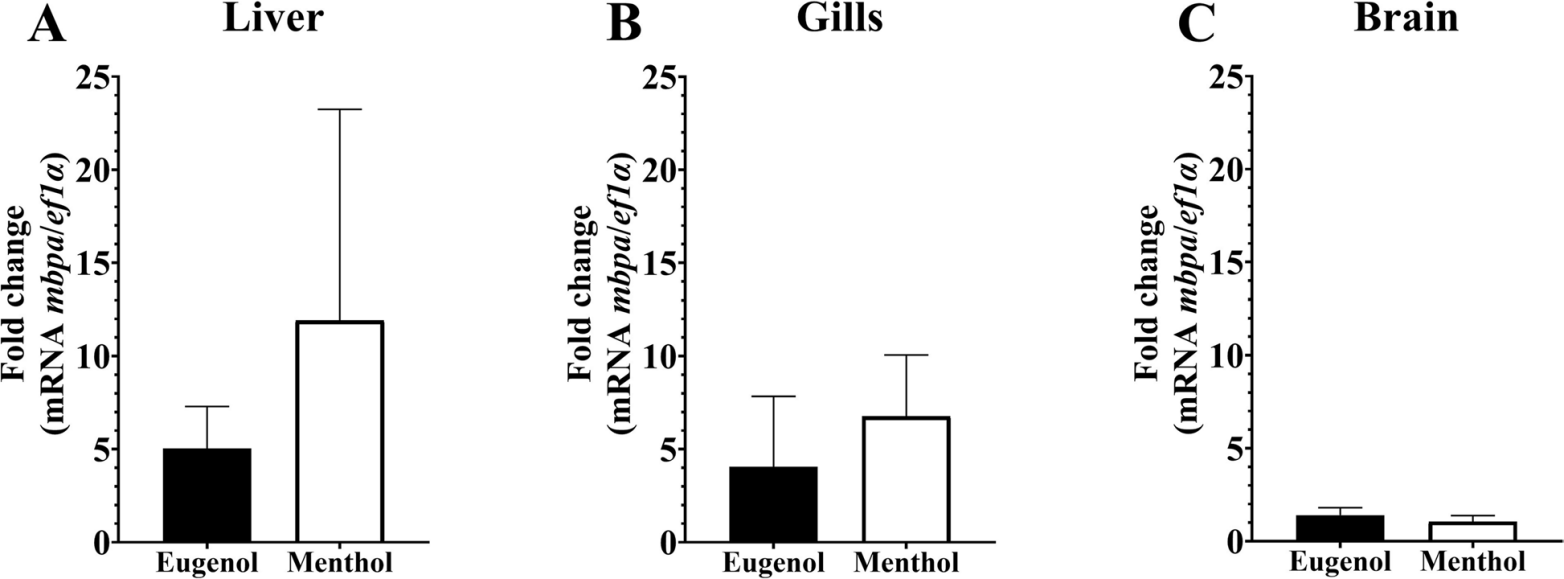
Relative gene expression of *mbpa* transcripts in *Piaractus brachypomus* under anesthesia with eugenol (40 mg/L) and menthol (50 mg/L). **A** *mbpa* levels in liver tissue; **B** *mbpa* levels in gill tissue; **C** *mbpa* levels in brain tissue. *ef1a* was used as a reference gene. *p < 0.05

**Fig. 6:**
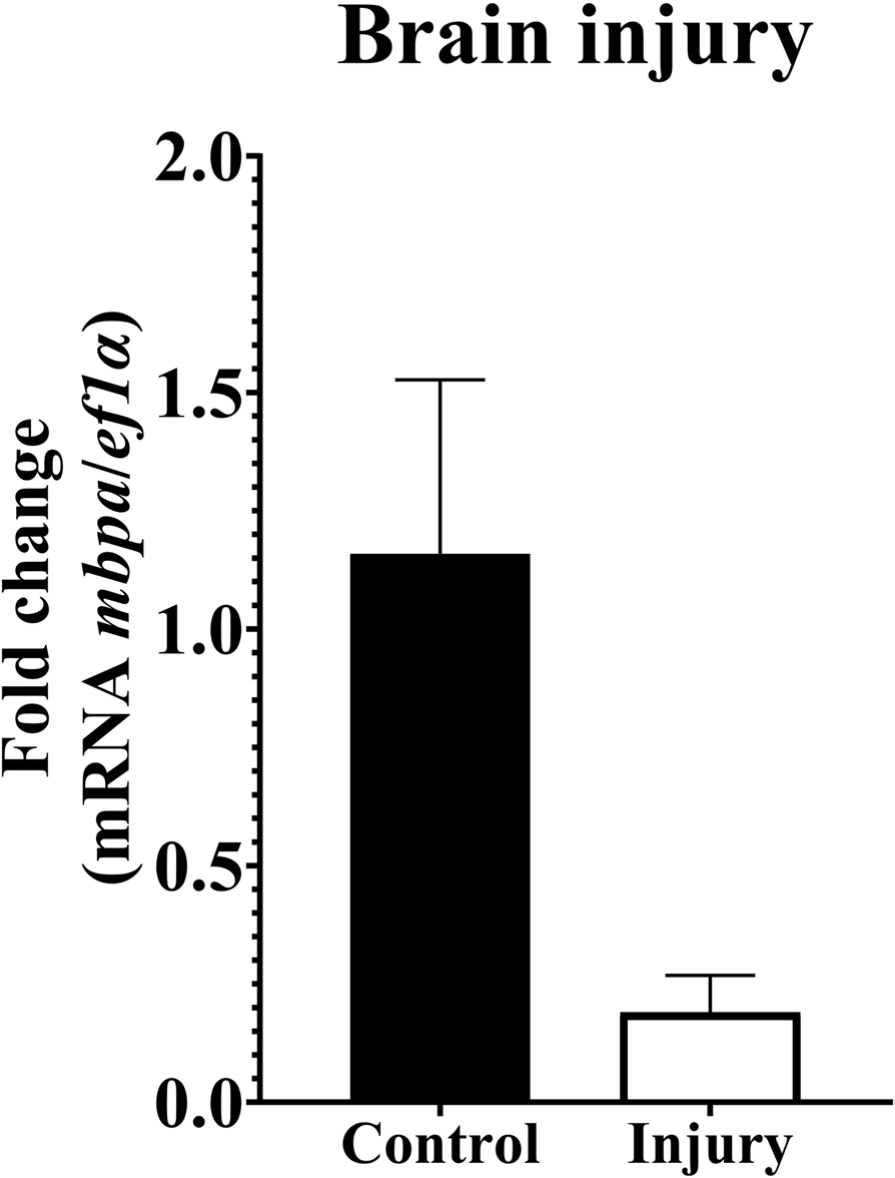
Relative gene expression of *mbpa* transcripts in brain tissues of *Piaractus brachypomus* after an injury. *ef1a* was used as a reference gene. *p < 0.05

The expression of *mbpa* gene in the optic chiasm showed significant differences, where it was upregulated in fish exposed to chlorpyrifos (*p* < 0.015). Nevertheless, in the olfactory bulb and telencephalon, the mRNA level of *mbpa* showed no significant differences (Fig. 4). Additionally, *mbpa* mRNA levels in the liver, gills, and brain tissues showed no significant differences in the fishes under anesthesia with menthol or eugenol (Fig. 5). Similarly, no statistical difference was observed in *mbpa* transcripts among the control group and brain injury group (Fig. 6).

## Discussion

### MBPa protein characterization

Previously, MBPa and MBPb isoforms have been reported in several fishes due to genomic duplications [1]. In this study, MBPa from *P*. *brachypomus* was identified as 137 aa protein encoded by 414 nucleotides, such as the proteins of *Cyprinus carpio* (XP_018938868.1), *Colossoma macropomum* (XP_036423499.1), *Electrophorus electricus* (XP_026875339.1), and *Pygocentrus nattereri* (XP_017556099.1). In addition, *P*. *brachypomus* MBPa showed a molecular weight of 15.5 kDa, which agrees with the size of MBPa from *Danio rerio* detected by 1D-SDS-PAGE, ranging from 12 to 20 kDa [1]. Similarly, in mammals, MBP isoforms exhibit molecular weight between 14 and 21.5 kDa, of which the isoforms 18.5 kDa and 14 kDa are the most common [7, 36]. On the other hand, computed theoretical pI was 12.28, closely to the reported for MBP, recognized as a basic protein with an isoelectric point of 10.8 [37]. This characteristic pI is crucial to the MBP binding ability to acidic lipids on the neuronal surface since in the brain intracellular pH is maintained at approximately 7.2, which gives it a predominantly positive charge to the protein [37, 38]. Furthermore, it has been reported the loss of these electrostatic interactions at a pH value of 10.8, due to MBP acquiring a neutral charge [37]. MBPa from *P. brachypomus* showed a greater proportion of positively charged residues, an important property considering that basic/positive motifs (Arg+Lys) of MBP allow it to bind and interact with negatively charged lipids as phosphatidylinositol 4,5-bisphosphate (PIP2) [1, 39]. It has been suggested that this interaction is responsible to pull together the cytoplasmic surfaces of adjacent myelin membranes and for the myelin biogenesis [1, 40]. Also, the association with negative lipids and positive MBP amino acids represents a conserved feature of myelin, being widely distributed among vertebrates as mammals, cartilaginous, and teleost fish [1, 39]. The instability index obtained classified MBPa of our study as unstable protein on in vitro conditions according to the scale described by Guruprasad et al. [41]. However, in vivo, the interaction of the MBP with phospholipids and galactosylceramides contributes to myelin sheath formation, stability, and functions, turning myelin as one of the most stable anatomical components [1, 42–44]. The computed GRAVY index was negative (−1.036), on a scale from 2 to −2 [45], indicating that MBPa has principally a hydrophilic nature. Regarding this, MBP has been defined as hydrophilic protein, where regions of hydrophilic character have a high propensity to the formation of β-turns and coil [46, 47]. On the other hand, MBPa was predicted with 99 from 137 amino acids that form IDRs, being the 72.26% of whole protein, which is consistent with Harauz et al. [48] who reported that MBP has a high proportion of these regions, with around 75% of a random coil. In the same way, MBP is described as intrinsically disordered protein, due to some features described above as its high positive charge and low hydrophobicity gives structural flexibility with a large effective surface area that adapts to several interactions with different binding partners and surfaces, but still specifically [7, 11, 48].

It is important to highlight that several domains and functional motifs have been recognized for MBP from mammals [49], corresponding to Ca^2+^-dependent calmodulin-binding regions both in N-terminal than C-terminal region [50], ligand of an SH3 (Src homology 3) domain [51], MAP-kinase binding sites [48, 52], β-strands conserved sequences [53], and immunodominant region [54]. Also, the post-translational modifications of MBP protein for mammals have been completely characterized [48]. Nevertheless, in the MBPa described in this study, any of these regions were found, similarly to the report by Nawaz et al. [1] who report no evidence for *golli* peptides in the *mbpa* gene or transcripts for *Danio rerio* isoforms. This could be explained due to MBPa in fish which is not being encoded by alternative splicing from Golli-MBP, but is encoded by a distinct gene with a different genomic location [1]. Therefore, it is possible to suggest that functional regions have diverged among teleost and other vertebrates based on the available information [1, 49]; in spite of several domains and motifs that must be present in MBPa from fish, the lack of functional and structural information hinders its identification and modeling.

### Phylogenetic analysis

Our results of protein sequence identity from the alignment of MBP isoforms are according to Nawaz et al. [1] who found similarity from 22 to 99% in MBP from fish and suggest these values as the outcome of a low selective pressure to the evolutive maintenance of the particular amino acid sequences. The distribution of the taxons in the tree reflects the known MBP isoforms and evolutive relationships in fish. In this way, Nawaz et al. [1] showed a unique MBP for cartilaginous fish, while two isoforms have been recognized from teleost fish. The cartilaginous fish group, represented by rays and sharks, is remarkably separated from teleost clades. This may be explained due to the fact that myelin was originated in jawed vertebrates, since superclass Gnathostomata and the consequently evolutive radiation to tetrapods, with the chondrichthyes fishes being the most ancient living vertebrates with multilayered and compacted myelin sheath [55]. In addition, MBP from cartilaginous fish is more closely related to MPB from tetrapods than MBP found in teleost fish [47, 49].

It is important to note that the 4 teleost species with both MBP isoforms were distributed according to MBPa and MBPb clades, suggesting a divergence among these proteins. Regarding this, it has been reported differential expression of these variants in the embryonic development of zebrafish with a functional specialization [1].

### Tissue basal expression of mbpa

In this work, the high expression of *mbpa* mRNA in brain tissues was expected, due to MBP being widely distributed in the central nervous system by the action of the oligodendrocytes [4]. The presence of *mbpa* transcripts found in different normal tissues agrees with the well-known distribution of MBP in the peripheral nervous system, and its isoforms described in blood cell lineages, immune tissues like lymph nodes, and macrophages [56–58]. About this, the expression of MBP transcripts in the immune system may be important for self-tolerance to CNS, as stated by Kalwy et al. [57]. Additionally, it has been reported that MBP isoforms have a differential location in the cells and myelin, which suggests that they could have distinct functions [7]. It is important to highlight that we found the expression of *mpba* in both CNS and PNS-related tissues, in contrast with the expression of this specific isoform reported by Torvund-Jensen et al. [59], who showed that *mpba* is restricted to the PNS in *Danio rerio*, which could indicate a functional divergence of MBP isoforms among fishes.

On the other hand, the significantly higher expression of *mbpa* mRNA in optic chiasm than olfactory bulb may be explained due to the fact that some brain zones require a greater neuronal transmission capacity, for which the myelin composition is variable within this organ according to the differential functions [60]. Thus, the olfactory nerve is composed of unmyelinated axons, while the optic nerve axons are myelinated by oligodendrocytes, in the same form as those in the white matter tracts of the brain and the spinal cord [60, 61]. Therefore, our results agree with the anatomical distribution and physiological functions of the myelin in both brain regions.

### Gene expression of mbpa in sublethal chlorpyrifos exposition

CPF is an organophosphate widely used in the world in crops and it has been related to both human and animal health risks including several physiological disorders at neurological, endocrine, immune, hematological, and reproductive levels [62]. Previous studies show autoimmunity targeting myelin as a result of CPF exposure and abnormalities in the distribution and formation of the myelin due to similar organophosphate compounds [63, 64]. In the same way, Holguín et al. [18] reported encephalic lesions in *P. brachypomus* after sublethal CPF exposure (0.011 μg/L). Due to its key role as constitutive protein and for neuronal transmission [1, 7], we suggest that *mbpa* mRNA was upregulated to provide protection against neurotoxicity induced by CPF. It is important to note that the optic nerve and telencephalon are myelinated, while olfactory nerves present axons without myelin [61, 65, 66]. Likewise, El-Hossary et al. [67] showed that CPF causes abnormal myelin sheath, which in turn degenerates optic nerves. On the other hand, several studies have determined that induced neurotoxicity can generate demyelination between 3 and 4 weeks after exogenous substance exposition [68, 69], and remyelination may begin 1 or 2 months after [70]. Additionally, CPF with aberrant myelin form due to events triggered after chemical exposure such as alterations in the MBP expression, reduction in myelin-associated glycoprotein (MAG) mRNA, and viability of oligodendrocytes has been related [71–73]. According to this, *mbpa* could be expressed in an anticipatory and exacerbated way in the optic chiasm from *P*. *brachypomus*, causing abnormal myelin. However, in this study, we do not analyze the morphology of myelin in CPF exposed fishes; therefore, it is suggested to evaluate this aspect in further studies.

### Gene expression of mbpa in anesthesia assay

Anesthesia is widely used in fishes to reduce stress and guarantee the welfare of animals during surgery and aquaculture procedures as handling, transport, and feeding as well as in biological research [74, 75]. In fish, anesthesia is administered by immersion; the anesthetic compound is ventilated from a solution through the gills and/or absorbed for the skin, then enters the bloodstream, and is transported to the CNS [76]. As general anesthetics, both eugenol and menthol suppress CNS activity and the outcome is a state of unconsciousness with a total lack of movement and sensation, referred to as deep anesthesia [32, 75]. Regarding these compounds, Zapata et al. [32] demonstrated rapid induction and recovery times at low doses, without mortality or any apparent physiological alterations in *P*. *brachypomus*. Additionally, previous studies demonstrated reversible changes on myelin structure during anesthesia, proportional to the duration and concentration of the used compound, that disappear upon removal of the anesthetic [77]. Also, a high expression of *mbp* in zebrafish anesthetized using propofol, showing its transcripts as an indicator of the effect of exogenous compounds in the central nervous system, has been reported [13]. Therefore, the lack of deregulation of *mbpa* gene expression found in this study reinforces the idea of no negative effects long-term on general physiological conditions and in the structure of the nervous system of this species after anesthesia with menthol and eugenol.

### Gene expression of mbpa in acute brain injury

TBI is a major global health issue, affecting millions of individuals around the world and with an increasing number of cases in recent years [78]. For that reason, research of rapidity available biomarkers for robust diagnosis as well as prognosis of TBI have a growing interest [15]. Studies suggest MBP as a biomarker of TBI due to its neuro-specific role in myelin [14, 46]. In addition, fish could be used as a model organism for evaluating TBI because of its robust physiological response [79], as assessed in zebrafish (*Danio rerio*) [80, 81]. In this study, we use *mbpa* as a marker of myelination, finding no changes in its gene expression after acute brain injury. Regarding this, Taib et al. [82] report in mice an increase in gene expression of protein-related myelin after 6 h post-injury, suggesting a protective response in the brain due to the myelin loss after mechanical damage. Similarly, Mierzwa et al. [83] showed significant demyelination at 3 days after induced TBI, followed by remyelination at 1 week. Thus, it is possible to suggest that after 2 h the regeneration processes of myelin are too early to be detected.

Regarding MBP as a biomarker of brain damage, Halstrom et al. [14] report MPB concentrations elevated in the plasma of patients with TBI compared to controls. Notably, MBP levels in serum as a biomarker of TBI present a high specificity (96%), but a low sensitivity (44%) [84]. Additionally, it is released late into blood (48 to 72 h post-injury), making it temporally unfavorable [85]. It is important to note that we do not measure MBPa in serum, which could be taken into account in conjunction with analysis of acute and/or chronic brain injury for future studies.

## Conclusions

The information obtained through amino acid bioinformatic analysis agrees with the molecular interactions and physiological functions of myelin basic proteins reported before and its basal gene expression is according to the anatomical distribution in different tissues analyzed. The high gene expression of *mbpa* in the brain of the CPF group indicated an adaptive response to protect against neurotoxicity and could be used as a biomarker of cerebral damage induced by chemical compounds. In the case of the lack of *mbpa*, mRNA level changes after anesthesia with menthol and eugenol indicate no negative long-term effects on physiological state and in the nervous system of this species, demonstrating the safety of these compounds as fish anesthetics. On the other hand, the gene expression of *mbpa* in the acute brain injury model showed that remyelination is a late process in *P*. *brachypomus* and does not occur within 2 h of post-lesion. More studies are necessary to expand the knowledge regarding functional domains and post-translational modifications as well as the tertiary structure of the MBPa from *P*. *brachypomus*. To the author’s knowledge, this is the first molecular characterization of the myelin basic protein a (*mbpa*) gene from the Colombian native species red-bellied pacu (*Piaractus brachypomus*).

## Data Availability

The authors declare that all generated and analyzed data are included in the article.

## Abbreviations

*mbpa*: Myelin basic protein a gene
*ef1α*: Elongation factor 1-α gene
ORF: Open reading frame
MBP: Myelin basic protein
MBPa: Myelin basic protein a
MBPs: Myelin basic proteins
CNS: Central nervous system
PNS: Peripheral nervous system
mRNA: Messenger RNA
cDNA: DNA complementary to RNA
CPF: Chlorpyrifos
TBI: Traumatin brain injury
PCR: Polymerase chain reaction
ddH_2_O: Distilled deionized water
BLASTn: Nucleotide-nucleotide BLAST
BLASTp: Protein-protein BLAST
IDRs: Intrinsically disordered regions
MSA: Multiple sequence alignment

## References

[1] Nawaz S, Schweitzer J, Jahn O, Werner HB (2013). Molecular evolution of myelin basic protein, an abundant structural myelin component. GLIA.

[2] Watanabe T, Watanebe T (2018). The Cell. Biophysical basis of physiology and calcium signaling mechanism in cardiac and smooth muscle.

[3] Yergert KM, Doll CA, O’Rouke R, Hines JH, Appel B (2021). Identification of 3′ UTR motifs required for mRNA localization to myelin sheaths *in vivo*. PLoS Biol.

[4] Baraban M, Mensch S, Lyons DA (2016). Adaptive myelination from fish to man. Brain Res.

[5] Kirschner DA, Karthigesan J, Bizzozero OA, Kosaras B, Inouye H (2008). Myelin structure and composition of myelinated tissue in the African lungfish. Neuron Glia Biol.

[6] Kumar S, Sharma B, Bhadwal P, Sharma P, Agnihotri N, Holban AM, Grumezescu AM (2018). Lipids as nutraceuticals: a shift in paradigm. Handbook of food bioengineering, therapeutic foods.

[7] Boggs JM (2006). Myelin basic protein: a multifunctional protein. Cellular Mol Life Sci CMLS.

[8] Brösamle C, Halpern ME (2002). Characterization of myelination in the developing zebrafish. Glia.

[9] Zhou L, Li CJ, Wang Y, Xia W, Yao B, Jin JY, Gui JF (2007). Identification and characterization of a MBP isoform specific to hypothalamus in orange-spotted grouper (*Epinephelus coioides*). J Chem Neuroanat.

[10] Steshenko O, Andrade DM, Honigmann A, Mueller V, Schneider F, Sezgin E, Hell SW, Simons M, Eggeling C (2016). Reorganization of lipid diffusion by myelin basic protein as revealed by STED nanoscopy. Biophys J.

[11] Harauz G, Ladizhansky V, Boggs JM (2009). Structural polymorphism and multifunctionality of myelin basic protein. Biochemistry.

[12] Zuchero JB, Fu MM, Sloan SA, Ibrahim A, Olson A, Zaremba A, Dugas JC, Wienbar S, Caprariello AV, Kantor C (2016). CNS myelin wrapping is driven by actin disassembly. Dev Cell.

[13] D’ Amora M., Giordani S. (2018) The utility of zebrafish as a model for screening developmental neurotoxicity. Front Neurosci 12. 10.3389/fnins.2018.00976 10.3389/fnins.2018.00976 PMC6305331 30618594

[14] Halstrom A, MacDonald E, Neil C, Arendts G, Fatovich D, Fitzgerald M (2017). Elevation of oxidative stress indicators in a pilot study of plasma following traumatic brain injury. J Clin Neurosci.

[15] Mehta T, Fayyaz M, Giler GE, Kaur H, Raikwar SP, Kempuraj D, Selvakumar GP, Ahmed ME, Thangavel R, Zaheer S (2020). Current trends in biomarkers for traumatic brain injury. Open Access J Neurol Neurosurg.

[16] Schartl M (2014). Beyond the zebrafish: diverse fish species for modeling human disease. Dis Model Mech.

[17] Lust K, Tanaka EM (2019). A comparative perspective on brain regeneration in amphibians and teleost fish. Dev Neurobiol.

[18] Holguín-Céspedes GK, Millán-Ocampo LM, Mahecha-Méndez EJ, Céspedes-Rubio ÁE, Rondón-Barragán IS (2019). Toxicity assessment of chlorpyrifos in red-bellied pacu fingerlings (*Piaractus brachypomus*). Revista Internacional de Contaminación Ambiental.

[19] Marín-Mendez G, Torres-Cortes A, Naranjo-Suarez L, Chacón-Novoa R, Rondón-Barragan I (2012). Concentración letal 50 a 96 horas de eugenol en cachama blanca (*Piaractus brachypomus*). ORINOQUIA.

[20] Mesa-Granda M, Botero-Aguirre M (2007). La cachama blanca (*Piaractus brachypomus*), una especie potencial para el mejoramiento genético. Revista Colombiana de Ciencias Pecuarias.

[21] Naranjo-Gómez JS, Vargas-Rojas LF, Rondón-Barragán IS (2013). Toxicidad aguda de cloruro de mercurio (HGCL2) en Cachama blanca: *Piaractus brachypomus* (Cuvier, 1818). Actualidades Biológicas.

[22] Brattelid T, Smith AJ (2000). Methods of positioning fish for surgery or other procedures out of water. Lab Anim.

[23] Jenkins JA, Chair HL, Bart J, Bowker JD, Bowser PR, MacMillan JR, Nickum JG, Rose JD, Sorensen PW, Whitledge GW, Rachlin JW, Warkentine BE, Bart HL (2014). Guidelines for the use of fishes in research.

[24] Kearse M, Moir R, Wilson A, Stones-Havas S, Cheung M, Sturrock S, Buxton S, Cooper A, Markowitz S, Duran C (2012). Geneious Basic: an integrated and extendable desktop software platform for the organization and analysis of sequence data. Bioinformatics.

[25] Blum M, Chang HY, Chuguransky S, Grego T, Kandasaamy S, Mitchell A, Nuka G, Paysan-Lafosse T, Qureshi M, Raj S (2021). The InterPro protein families and domains database: 20 years on. Nucleic Acids Res.

[26] Necci M, Piovesan D, Dosztányi Z, Tosatto SC (2017). MobiDB-lite: fast and highly specific consensus prediction of intrinsic disorder in proteins. Bioinformatics.

[27] Gasteiger E, Hoogland C, Gattiker A, Wilkins MR, Appel RD, Bairoch A, Walker JM (2005). Protein identification and analysis tools on the ExPASy server. The proteomics protocols handbook.

[28] Saitou N, Nei M (1987). The neighbor-joining method: a new method for reconstructing phylogenetic trees. Mol Biol Evol.

[29] Jukes TH, Cantor CR (1969). Evolution of protein molecules. Mammalian Prot Metab.

[30] Vargas-Vargas RA (2017) Pez cebra (*Danio rerio*) y anestesia. Un modelo animal alternativo para realizar investigación biomédica básica. Anestesia en México (29):86–96 http://www.scielo.org.mx/pdf/am/v29s1/2448-8771-am-29-00086.pdf

[31] CCAC (2010). CCAC guidelines on: Euthanasia of animals used in science.

[32] Zapata-Guerra NA, Rueda-Gómez DS, Lozano-Villegas KJ, Herrera-Sánchez MP, Uribe-García HF, Rondón-Barragán IS (2020). Menthol as anaesthetic for red-bellied pacu (*Piaractus brachypomus*) and its effect on HIF1a and GlucoR gene expression. Aquac Res.

[33] Kishimoto N, Shimizu K, Sawamoto K (2012). Neuronal regeneration in a zebrafish model of adult brain injury. DMM Dis Models Mechan.

[34] Schmidt R, Beil T, Strähle U, Rastegar S (2014). Stab wound injury of the zebrafish adult telencephalon: a method to investigate vertebrate brain neurogenesis and regeneration. J Vis Exp.

[35] Livak KJ, Schmittgen TD (2001). Analysis of relative gene expression data using real-time quantitative PCR and the 2-ΔΔCT method. Methods.

[36] Ridsdale RA, Beniac DR, Tompkins TA, Moscarello MA, Harauz G (1997). Three-dimensional structure of myelin basic protein: II. Molecular modeling and considerations of predicted structures in multiple sclerosis. J Biol Chem.

[37] Zhang J, Sun X, Zheng S, Liu X, Jin J, Ren Y, Luo J (2014). Myelin basic protein induces neuron-specific toxicity by directly damaging the neuronal plasma membrane. PLoS One.

[38] Casey JR, Grinstein S, Orlowski J (2010). Sensors and regulators of intracellular pH. Nat Rev Mol Cell Biol.

[39] Nawaz S, Kippert A, Saab AS, Werner HB, Lang T, Nave KA, Simons M (2009). Phosphatidylinositol 4, 5-bisphosphate-dependent interaction of myelin basic protein with the plasma membrane in oligodendroglial cells and its rapid perturbation by elevated calcium. J Neurosci.

[40] Min Y, Kristiansen K, Boggs JM, Husted C, Zasadzinski JA, Israelachvili J (2009). Interaction forces and adhesion of supported myelin lipid bilayers modulated by myelin basic protein. Proc Natl Acad Sci.

[41] Guruprasad K, Reddy BB, Pandit MW (1990). Correlation between stability of a protein and its dipeptide composition: a novel approach for predicting in vivo stability of a protein from its primary sequence. Protein Eng Des Sel.

[42] O'Brien JS (1965). Stability of the myelin membrane: lipid molecules may impart stability to the myelin membrane through intermolecular cohesion. Science.

[43] Poitelon Y, Kopec AM, Belin S (2020). Myelin fat facts: an overview of lipids and fatty acid metabolism. Cells.

[44] Valdivia AO, Agarwal PK, Bhattacharya SK (2020). Myelin basic protein phospholipid complexation likely competes with deimination in experimental autoimmune encephalomyelitis mouse model. ACS Omega.

[45] Kyte J, Doolittle RF (1982). A simple method for displaying the hydropathic character of a protein. J Mol Biol.

[46] Glushakova OY, Glushakov AV, Mannix R, Miller ER, Valadka AB, Hayes RL. 2018. The use of blood-based biomarkers to improve the design of clinical trials of traumatic brain injury. In Skolnick BE, Alves WM. (Eds.), Handbook of neuroemergency clinical trials. Elsevier: Academic, pps. 139-166. 10.1016/B978-0-12-804064-5.00008-4

[47] Saavedra RA, Fors L, Aebersold RH, Arden B, Horvath S, Sanders J, Hood L (1989). The myelin proteins of the shark brain are similar to the myelin proteins of the mammalian peripheral nervous system. J Mol Evol.

[48] Harauz G, Ishiyama N, Hill CM, Bates IR, Libich DS, Fares C (2004). Myelin basic protein-diverse conformational states of an intrinsically unstructured protein and its roles in myelin assembly and multiple sclerosis. Micron.

[49] Harauz G, Libich DS (2009). The classic basic protein of myelin-conserved structural motifs and the dynamic molecular barcode involved in membrane adhesion and protein-protein interactions. Curr Protein Pept Sci.

[50] Bamm VV, De Avila M, Smith GS, Ahmed MA, Harauz G (2011). Structured functional domains of myelin basic protein: cross talk between actin polymerization and Ca2+-dependent calmodulin interaction. Biophys J.

[51] Polverini E, Rangaraj G, Libich DS, Boggs JM, Harauz G (2008). Binding of the proline-rich segment of myelin basic protein to SH3 domains: spectroscopic, microarray, and modeling studies of ligand conformation and effects of posttranslational modifications. Biochemistry.

[52] Hirschberg D, Rådmark O, Jörnvall H, Bergman T (2003). Thr94 in bovine myelin basic protein is a second phosphorylation site for 42-kDa mitogen-activated protein kinase (ERK2). J Protein Chem.

[53] Stoner GL (1990). Conservation throughout vertebrate evolution of the predicted β-strands in myelin basic protein. J Neurochem.

[54] Wucherpfennig KW, Catz I, Hausmann S, Strominger JL, Steinman L, Warren KG (1997). Recognition of the immunodominant myelin basic protein peptide by autoantibodies and HLA-DR2-restricted T cell clones from multiple sclerosis patients. Identity of key contact residues in the B-cell and T-cell epitopes. J Clin Invest.

[55] Bullock TH, Moore JK, Fields RD (1984). Evolution of myelin sheaths: both lamprey and hagfish lack myelin. Neurosci Lett.

[56] Liu HB, MacKenzie-Graham AJ, Palaszynski K, Liva S, Voskuhl RR (2001). “Classic” myelin basic proteins are expressed in lymphoid tissue macrophages. J Neuroimmunol.

[57] Kalwy S, Marty MC, Bausero P, Pessac B (1998). Myelin basic protein-related proteins in mouse brain and immune tissues. J Neurochem.

[58] Marty MC, Alliot F, Rutin J, Fritz R, Trisler D, Pessac B (2002). The myelin basic protein gene is expressed in differentiated blood cell lineages and in hemopoietic progenitors. Proc Natl Acad Sci.

[59] Torvund-Jensen J, Steengaard J, Askebjerg LB, Kjaer-Sorensen K, Laursen LS (2018). The 3’UTRs of myelin basic protein mRNAs regulate transport, local translation and sensitivity to neuronal activity in zebrafish. Front Mol Neurosci.

[60] Gandhi S, Abramov AY (2012) Mechanism of oxidative stress in neurodegeneration. Oxidative Med Cell Longev 2012. 10.1155/2012/428010 10.1155/2012/428010 PMC3362933 22685618

[61] Tarulli A (2021). Disorders of the eyelids and pupils. Neurology.

[62] ur Rahman HU, Asghar W, Nazir W, Sandhu MA, Ahmed A, Khalid N (2020) A comprehensive review on chlorpyrifos toxicity with special reference to endocrine disruption: evidence of mechanisms, exposures and mitigation strategies. Sci Total Environ 142649. 10.1016/j.scitotenv.2020.142649 10.1016/j.scitotenv.2020.142649 33059141

[63] Tapiero-Hernández Y, Rondon-Barragán I, Cespedes-Rubio A (2013). Neurotoxic potential of trichlorfon to multiple sublethal doses in wistar rats. Acta Biológica Colombiana.

[64] Thrasher JD, Madison R, Broughton A (1993). Immunologic abnormalities in humans exposed to chlorpyrifos: preliminary observations. Arch Environ Health Int J.

[65] García-Gonzalez D, Murcia-Belmonte V, Clemente D, De Castro F (2013). Olfactory system and demyelination. Anat Rec.

[66] Haines DE, Mihailoff GA, Haines DE, Mihailoff GA (2018). Chapter 16 - the telencephalon. Fundamental neuroscience for basic and clinical applications (fifth edition).

[67] El-Hossary GG, Mansour SM, Mohamed AS (2009) Neurotoxic effects of chlorpyrifos and the possible protective role of antioxidant supplements: an experimental study. J Appl Sci Res 5(9):1218–1222

[68] Pott F, Gingele S, Clarner T, Dang J, Baumgartner W, Beyer C, Kipp M (2009). Cuprizone effect on myelination, astrogliosis and microglia attraction in the mouse basal ganglia. Brain Res.

[69] Millet V, Marder M, Pasquini LA (2012). Adult CNP: EGFP transgenic mouse shows pronounced hypomyelination and an increased vulnerability to cuprizone-induced demyelination. Exp Neurol.

[70] Hanafy KA, Sloane JA (2011). Regulation of remyelination in multiple sclerosis. FEBS Lett.

[71] Betancourt AM, Burgess SC, Carr RL (2006). Effect of developmental exposure to chlorpyrifos on the expression of neurotrophin growth factors and cell-specific markers in neonatal rat brain. Toxicol Sci.

[72] Garcia SJ, Seidler FJ, Slotkin TA (2003). Developmental neurotoxicity elicited by prenatal or postnatal chlorpyrifos exposure: effects on neurospecific proteins indicate changing vulnerabilities. Environ Health Perspect.

[73] Slotkin TA, Seidler FJ (2007). Comparative developmental neurotoxicity of organophosphates in vivo: transcriptional responses of pathways for brain cell development, cell signaling, cytotoxicity and neurotransmitter systems. Brain Res Bull.

[74] Priborsky J, Velisek J (2018). A review of three commonly used fish anesthetics. Rev Fisheries Sci Aquacult.

[75] Martins T, Valentim A, Pereira N, Antunes LM (2019). Anaesthetics and analgesics used in adult fish for research: a review. Lab Anim.

[76] Neiffer DL, Stamper MA (2009). Fish sedation, anesthesia, analgesia, and euthanasia: considerations, methods, and types of drugs. ILAR J.

[77] Mateu L, Moran O, Padrón R, Borgo M, Vonasek E, Marquez G, Luzzati V (1997). The action of local anesthetics on myelin structure and nerve conduction in toad sciatic nerve. Biophys J.

[78] Galgano M, Toshkezi G, Qiu X, Russell T, Chin L, Zhao LR (2017). Traumatic brain injury: current treatment strategies and future endeavors. Cell Transplant.

[79] Cho SJ, Park E, Telliyan T, Baker A, Reid AY (2020). Zebrafish model of posttraumatic epilepsy. Epilepsia.

[80] Cacialli P, D'angelo L, Kah O, Coumailleau P, Gueguen MM, Pellegrini E, Lucini C (2018). Neuronal expression of brain derived neurotrophic factor in the injured telencephalon of adult zebrafish. J Comp Neurol.

[81] Maheras AL, Dix B, Carmo OMS, Young AE, Gill VN, Sun JL, Booker AR, Thomason HA, Ibrahim AE, Stanislaw L et al (2018) Genetic pathways of neuroregeneration in a novel mild traumatic brain injury model in adult zebrafish. ENeuro 5(1). 10.1523/ENEURO.0208-17.2017 10.1523/ENEURO.0208-17.2017 PMC5752677 29302617

[82] Taib T, Leconte C, Van Steenwinckel J, Cho AH, Palmier B, Torsello E, Kuen RL, Onyeomah S, Ecomard K, Benedetto C (2017). Neuroinflammation, myelin and behavior: temporal patterns following mild traumatic brain injury in mice. PLoS One.

[83] Mierzwa AJ, Marion CM, Sullivan GM, McDaniel DP, Armstrong RC (2015). Components of myelin damage and repair in the progression of white matter pathology after mild traumatic brain injury. J Neuropathol Exp Neurol.

[84] Berger RP, Adelson PD, Pierce MC, Dulani T, Cassidy LD, Kochanek PM (2005). Serum neuron-specific enolase, S100B, and myelin basic protein concentrations after inflicted and noninflicted traumatic brain injury in children. J Neurosurg Pediatr.

[85] Kim HJ, Tsao JW, Stanfill AG (2018) The current state of biomarkers of mild traumatic brain injury. JCI insight 3(1). 10.1172/jci.insight.97105 10.1172/jci.insight.97105 PMC5821170 29321373

